# Primary Prevention of Lead Exposure—Blood Lead Results at Age Two Years

**DOI:** 10.3390/ijerph9041216

**Published:** 2012-04-11

**Authors:** Carla Campbell, Edward Gracely, Mary Tran, Naomi Starkey, Hans Kersten, Peter Palermo, Nancy Rothman, Laura Line, Tine Hansen-Turton

**Affiliations:** 1 School of Public Health, Drexel University, 1505 Race Street, MS 1034, Philadelphia, PA 19102, USA; Email: tranmary23@gmail.com; 2 College of Medicine, Family, Community, and Preventive Medicine, Drexel University, 2900 Queen Lane, Philadelphia, PA 19129, USA; Email: egracely@drexelmed.edu (E.G.); hans.kersten@drexelmed.edu (H.K.); 3 School of Medicine, University of California Davis, 4610 X Street, Sacramento, CA 95817, USA; 4 National Nursing Centers Consortium, 260 South Broad Street, Philadelphia, PA 19102, USA; Email: nstarkey@gmail.com (N.S.); rothman@temple.edu (N.R.); laural@rhd.org (L.L.); tine@nncc.us (T.H.-T.); 5 St. Christopher’s Hospital for Children, East Erie Avenue and North Front Street, Philadelphia, PA 19134, USA; 6 Philadelphia Department of Public Health, Childhood Lead Poisoning Prevention Program, 2100 West Girard Avenue, Building #3, Philadelphia, PA 19130, USA; Email: palermop2@yahoo.com

**Keywords:** childhood lead poisoning, primary prevention of lead exposure, blood lead levels, children’s environmental health

## Abstract

*Objectives*: The Philadelphia Lead Safe Homes (LSH) Study was designed to evaluate whether educational and environmental interventions in the first year of life for families of newborns increased knowledge of lead exposure prevention and were associated with less elevation of blood lead levels (BLLs) for these children, when compared to children receiving standard care. *Methods*: The current study performed descriptive statistics on the second-year BLL data for both groups and compared these using chi-square tests for proportions and unpaired t-tests for means. *Results*: A BLL result was found for 159 (50.6%) of the 314 LSH cohort children and 331 (52.7%) of the 628 control children (*p* = 0.1). Mean and standard deviation for age at draw was 23.8 (3.4) months *versus* 23.6 (3.1) months (*P* = 0.6). Geometric mean BLLs were 3.7 *versus* 3.5 µg/dL (*P* = 0.4). The percentages of the cohort group with a BLL of ≥20, ≥10 and ≥5 μg/dL, respectively, were 0.6%, 5% and 30%; for the controls 1.2%, 6.6%, and 25%. These percentages were not significantly different between groups. *Conclusion*: A comparison of geometric mean BLLs and percentages above several BLL cut points drawn at age two years in a group of urban newborns benefitting from study interventions *versus* a group of similar urban children did not yield statistically significant differences. Both groups had relatively lower lead levels when compared to historical cohort groups, which may reflect a continuing downward trend in BLLs in U.S. children. The interventions did result in benefits to the families such as an increase in parental knowledge about lead exposure prevention and in-home wet cleaning activity, and a decrease in lead dust levels in study homes.

## 1. Introduction

Primary prevention of lead exposure and lead poisoning in U.S. children has been recommended by the Centers for Disease Control and Prevention in several recent publications [1,2]. Prevention of lead exposure and elevated blood lead levels (BLLs) (level ≥10 micrograms per deciliter—µg/dL) has been increasingly endorsed due to the large number of studies documenting the adverse effects. These effects include various types of neuropsychological impairment impacting intelligence or IQ, academic performance, attention, development, and behavior; some studies suggest adverse effects at blood lead levels less than 10 µg/dL [2,3,4,5,6,7,8,9]. Lead has also been associated with many other adverse effects that include juvenile delinquency, anemia, effects on growth and the endocrine system, dental caries, and rarely, encephalopathy with or without death [2,4,5]. The main sources for exposure for U.S. children remain home-based lead hazards generated by deterioration of lead-based paint in pre-1978 housing and exposure to various products containing lead such as toys, consumer products, home remedies, foods and cosmetics [10,11]. Primary prevention involves prospectively preventing exposure to lead, such as preventing or remediating lead hazards in housing before any resident children are exposed and poisoned. One of the few studies of household-level primary prevention done previously was a randomized controlled trial of lead dust control in 275 urban children [12]. The effectiveness of dust control measures in preventing or limiting elevation of BLLs was then assessed. The intervention group received cleaning equipment, supplies and multiple (up to 8) visits with a lead dust control advisor *versus* no intervention for the control group. The authors found no differences in BLL or dust levels between groups. The baseline (6-month) geometric mean (GM) BLLs and 95% confidence intervals were 2.8 (2.5, 3.1) µg/dL in the intervention group *versus* 2.9 (2.7, 3.2) µg/dL in the control group and rose steadily in both groups. Levels at 12 months were 5.5 (4.9, 6.2) µg/dL and 5.9 (5.3, 6.6) µg/dL and peak BLLs at age 24 months were 7.3 (6.6, 8.2) µg/dL and 7.8 (6.9, 8.7) µg/dL, respectively. At 24 months 39 (31%) and 43 (36%) of intervention and control children had BLLs ≥10 µg/dL (*p* = 0.4), and 6 (5%) and 8 (7%) had BLLs ≥20 µg/dL (*p* = 0.5). A follow-up study at 48 months also found no difference in BLLs although GMs and 95% confidence intervals had decreased to 5.9 (5.3, 6.7) and 6.1 (5.5, 6.9) µg/dL, respectively [13]. A randomized control trial by Jordan *et al*. of 299 intervention subjects in Minneapolis, Minnesota aged 0-36 months who received 20 bi-weekly educational sessions on how to prevent lead exposure over one year with quarterly follow-up sessions for another 2 years compared them to 308 control children whose families only received lead poisoning prevention brochures [14]. BLLs were obtained every 4 months; for the 378 children with sufficient BLL data 23% developed BLLs >10 µg/dL before age 3 years; 81% of intervention subjects *versus* 73% (*p* = 0.08) of control subjects maintained BLLs under 10 µg/dL; and the intervention reduced the risk of a BLL ≥10 µg/dL by 34%. Mean BLLs were not reported [14]. Other previous studies were reviewed in our earlier paper and found little evidence that educational or other simple household-level interventions would reduce BLLs [15].

The Philadelphia Lead Safe Homes Study evaluated whether educational and environmental interventions implemented with families of newborns in the first year of life increased knowledge of lead exposure prevention and were associated with less elevation of blood lead levels (BLLs) for these children, when compared to children and their families receiving standard care without these preventive interventions. A recent publication detailed the findings of the Lead Safe Homes study to include research findings through the first year of life [15]. This publication gives updated findings regarding blood lead level analysis for levels drawn around two years of age. As the first year BLLs were drawn at the end of or soon after completion of the study interventions, the second year BLLs were of interest in assessing whether the educational and environmental interventions might have had a protective effect in leading to lower second year levels, even in the absence of ongoing contact with study staff. This was particularly important in determining the degree of intervention that might be required for prevention of lead exposure and elevated BLLs. 

## 2. Experimental Section

Study methods were detailed previously [15], and will be briefly summarized here. The study had two separate arms. One arm was the Lead Safe Homes study, a one-year randomized controlled trial for which families of newborns attending urban pediatric practices in Philadelphia were recruited to participate. Study interventions for all families in the LSH took place during three home visits starting at baseline soon after birth with a follow-up visit at 6 and 12 months. Educational materials stressing lead exposure prevention information and activities such as wet mopping and wet dusting for dust control using cleaning equipment provided were presented in detail at the first visit and reinforced at subsequent visits. Families were randomly assigned to receive standard lead education (standard education group or SEG) or standard education supplemented by specific home maintenance education (maintenance education group or MEG). This additional home maintenance supplemental material was the primary difference between the two groups and gave much detail on essential maintenance practices to keep a home in a lead safe condition. All homes were given a visual assessment and lead dust testing of three surfaces at baseline and 12 months. Most homes had some lead hazards and parents in either group were offered free lead remediation work conducted by a certified lead abatement contractor. Parental reports of blood lead level results were collected in the course of the study visits and supplemented by BLL data from both the subjects’ health care providers and the Childhood Lead Poisoning Prevention Program of the Philadelphia Department of Public Health (PDPH). Other data, including environmental and demographic data, were collected during the three study visits; these included 310 and 110 families for first and third visits, as considerable attrition of subjects occurred during the study. 

The other arm of the study was a comparison group for the LSH Study as a whole. Since all LSH families received substantial education and follow-up, it was of interest to compare them, as a whole, to a group of comparable children not given any study-specific interventions. A de-identified dataset of BLLs and ages within the electronic database of The Children’s Hospital of Philadelphia for children seen for primary care services was provided by the Pediatric Research Consortium for The Children’s Hospital of Philadelphia practices. This group served as the comparison group to the intervention groups, and was matched 2:1 by age, gender, census tract and racial/ethnic background with LSH cohort children. Eight children could not be matched on racial/ethnic background but were matched for the other characteristics. The research protocol was approved by the institutional review boards of the Philadelphia Department of Public Health, the Children’s Hospital of Philadelphia, the National Nursing Centers Consortium, St. Christopher’s Hospital for Children, and Drexel University.

Key study results published previously from this study [15] included significantly increased knowledge in both LSH randomized groups, which was retained over time, and a significant drop in lead dust levels for those two groups combined, with 36.9% above the EPA’s standards (250 µg/sq.ft. for windowsills and 40 µg/sq.ft. for floors) at baseline compared with 26.9% at 12 months, which was mostly due to a decrease in windowsill lead dust levels (*p* = 0.03). Although 274 (89.5%) and 58 (57.4%) families were referred for lead hazard remediation work at baseline and at 12 months, only twenty-eight percent (78 families) completed the process to achieve this. There were no other obvious lead sources identified, such as ceramics, folk remedies, cosmetics, foods, *etc*.; 4.8% had possible lead exposure from hobbies; and 39.8% of parents reported ever working in auto mechanics, battery plants, construction or other jobs potentially associated with lead exposure. A quarterly cleaning summary looked at wet dusting and wet mopping in key rooms of the home. Over the course of the study both the MEG and SEG groups improved their cleaning activity; the only significant difference was a higher percent with frequent mopping of dining rooms by SEG (44%), *versus* MEG (23%) families (*p* = 0.04). There were very few significant differences between the SEG and MEG groups [15]. 

A main study hypothesis tested in the current analysis was that interventions would lower the BLLs in the LSH study cohort when compared to the matched comparison group of children. In our report at one year, a significantly higher percent of LSH cohort children (88.9%; 279 children) had a BLL drawn around the first birthday compared to the control group (84.4%; 530 children) (*p* = 0.03). Geometric mean (GM) levels at age one year were 2.6 and 2.7, respectively, for the LSH cohort and control groups, and not significantly different (*p* = 0.5) [15]. The current study performed descriptive statistics on the second-year BLL data for both groups and compared proportions using chi-square tests and means using unpaired t-tests. To help normalize the blood lead levels, they were converted to logarithms (base 10). We intended to have blood lead levels measured at age one and two years but these were drawn by health care providers and not by study staff. As there was variability around the age at which the first and second blood lead levels were drawn, to increase clarity we will use the terms “year 1 BLL” to refer to the first BLL obtained and “year 2 BLL” as that drawn between 18 and 30 months of age. With the sample sizes achieved and an SD (standard deviation) of about 0.26, a mean difference of about 0.067 could be detected with 80% power 1-tailed in log (base 10) BLL, which corresponds to roughly a 0.5 to 0.6 difference in geometric mean BLLs. Therefore, the study was powered to detect fairly small differences in geometric mean levels.

In order to look for evidence for a difference in BLLs between the LSH cohort and the matched comparison group at two time points, we fitted two linear regression models. The first regression model aimed to evaluate the effect of the intervention on changes in BLL between birth and two years of age in the LSH group *versus* the control group. The dependent variable was year 2 BLL. In this analysis, we controlled for the exact age when the year 2 BLL was drawn (which varied within the window of 18–30 months of age) and type of health insurance (a proxy for socio-economic status) (See Appendix). 

The second regression model aimed to evaluate whether there was an additional effect of the intervention between the first and second BLL assessments. In this model, year 2 BLL was again the dependent variable, while year 1 BLL, the age at year 2 blood draw and type of health insurance were the predictor variables. This was fitted to a restricted dataset, as follows. In the original paper [15], we were flexible with dates and used all first-test results regardless of whether or not they were close to one year of age, but for the current comparison we wanted to clearly differentiate year 1 and year 2 data in order to determine whether changes between the year 1 BLLs and the year 2 BLLs differed between the LSH and control groups. Therefore, we restricted the year 1 BLLs to those that satisfied both of two conditions. To be used, the measurement must have been taken: (1) sometime during 6 to 17.9 months of age and (2) at least three months before the year 2 BLL we used (measured between ages 18 to 30 months) (See Appendix). 

A simpler comparison was also performed using an unpaired t-test to compare the two groups (LSH and comparison) on the mean changes in the log transformed BLLs between one and two years of age. The test was done by using an unpaired rather than a paired t-test because the match was a 2:1 match and there were many missing values for the year 2 BLLs for both the LSH and control children. 

## 3. Results

Table 1 illustrates the results of the new analysis. As shown in the table, there were no differences between the LSH and control children on the percentage who obtained a BLL result, the percentage of obtained results that were venous samples, or on mean BLL levels. 

In a separate analysis, when the data was limited to the 140 LSH cohort and 287 control children with data at both age points, there were no significant differences in geometric mean BLLs at age one year of 2.7 and 2.7 (*P* = 0.99), respectively, or at two years of 3.6 and 3.5 (*p* = 0.6), respectively. 

Table 1 also shows the ranges of BLLs in the two groups and a breakdown into intervals. Slightly more than a quarter of the children in the combined groups had a BLL ≥5 μg/dL. Although many fewer were at more clinically significant levels, we did see some children ≥10 μg/dL: 8/159 in the LSH group and 22/331 in the controls, slightly above 5% overall. One and four in the two groups, respectively, were ≥20 μg/dL, or about 1%. None of these percentages were significantly different between the groups. 

**Table 1:** Comparison of the Lead Safe Homes Study cohort and control group BLL values at the two year time point (from 18 to 30 months).

Characteristic	LSH Group	Control Group	*P*-Value
Maximum N	314	628	
N (%) with recorded BLL value	159 (50.6%)	331(52.7%)	>0.5 ^a^
BLL is venous specimen N (%)	151/158 (95.6%)	304/331 (91.8%)	0.1 ^a^
Mean age at draw (months) ± SD	23.8 ± 3.4	23.6 ± 3.1	0.6 ^b^
Median age at draw (months)	24.1	24.2	
Age range (months)	18–30	18–30	
Median blood lead (μg/dL)	3.2	3.3	
GM BLL, μg/dL (95% CI)	3.7 (3.4, 4.0)	3.5 (3.3, 3.7)	0.4 b
Range of BLLs (μg/dL)	(0.8–39.4)	(0.7–33.1)	
Percentages at different BLL levels			
≥5 µg/dL	47/159 (29.6%)	82/331 (24.8%)	0.3 a
≥10 µg/dL	8/159 (5.0%)	22/331 (6.6%)	0.5 a
≥20 µg/dL	1/159 (0.6%)	4/331 (1.2%)	0.9 c

BLLs = blood lead levels; CI = confidence interval; GM = geometric mean; LSH = Lead Safe Homes; SD = standard deviation; ^a^ *P*-values are two-tailed significance for uncorrected chi-square; ^b^ *P*-values are two-tailed significance for unpaired t test; ^c^ *P*-values are two-tailed significance for continuity corrected chi-square.

In our initial analysis, to maximize sample size, we used all “first” blood lead values available for the subjects; most, but not all, of which were within a window around 1 year of age. For the current analysis, we used levels within a 6-month window of 2 years (thus 18 to 30 months), using all available data within that window, even if it had been included in the analysis for the previous report. 

There was no evidence for a difference between the LSH cohort and the matched controls at time 2, regardless of control for other variables in a linear regression. Several different models were described above. None of these comparisons or regressions was even close to significance on the study group variable (See Appendix). Regression 1 showed that BLL was lower in those children with private health insurance, relative to Medicaid insurance. This significance was not seen in regression 2. Since the insurance type effect was significant in the model that did not control for year 1 BLL, we tested for an interaction between group and insurance type. The effect of insurance type remained significant (*p* = 0.03), but the interaction was not significant (*p* = 0.64). The geometric mean BLL was 0.94 lower in private patients *versus* Medicaid for the LSH group, and 0.62 lower in private patients *versus* Medicaid in the controls, a very similar pattern.

A simpler comparison for mean changes between the year 1 and year 2 by groups found this change to be 0.12 *versus* 0.11 (*p* = 0.6) for the LSH and control groups, respectively. Lastly, for the two-year BLL data geometric means for the SEG (3.9) and the MEG (3.5; *p* = 0.2) were not significantly different, nor for the MEG (3.5) when compared to the control group (3.5; *p* = 0.97). 

## 4. Discussion

Our study differed from the historical studies described in that it twice offered home evaluation and remediation of lead hazards, in addition to education and provision of cleaning instructions and supplies. While there were positive changes such as increased parental knowledge and cleaning activity and a decrease in elevated dust lead levels in the home seen with a year-long program involving educational and environmental interventions to prevent elevation of blood lead levels in urban newborn children, there were not significant differences in blood lead levels between the Lead Safe Homes cohort group *versus* a control group of similar urban children. 

We did see evidence for a lower year 2 BLL in the children covered by private insurance *versus* those with Medicaid, but this did not differ significantly by LSH *versus* control group (non-significant group by insurance interaction). One possible explanation for the overall finding is that families of children on private insurance may be able to maintain their homes in better condition.

Our geometric mean BLLs were in the 2 s at 12 months and 3 s at 24 months. What is interesting is that the Lanphear study results, described above and published in 1999 and 2000 [12,13] showed baselines lead levels in the 2 s at 6 months, in the 5 s at 12 months, in the 7 s at 24 months and in the 5–6 s at 48 months. Their 6-month levels compared with our 12 month results and then they demonstrated a steady increase to peak at 24 months, and slowly decline by 48 months. By contrast, the levels in our groups rose only slightly (in the 2 s to in the 3 s) by 24 months. This most likely reflects trends seen both nationally and in Philadelphia [15,16] within the last few decades of an overall decrease in BLLs in children. 

There might have been a sharper contrast between families receiving and not receiving interventions in a city whose lead poisoning prevention program is not as strong and active as the one in Philadelphia or in a city whose children have higher BLLs. The control families may have received lead exposure prevention from other sources that would have biased the blood lead comparison toward a null finding. National public health interventions such as banning lead from use in plumbing, from gasoline, from canned goods, and from use in residential housing starting in 1978 have all contributed to the lowering of BLLs in the U.S., and demolition of old, substandard properties has likely also contributed locally.

Our analysis found that 0.6% and 1.2%, 5% and 6.6%, 30% and 25% of the cohort and control groups had a BLL of ≥20, ≥10 and ≥5 μg/dL, respectively, which was not significantly different between groups. These were much lower percentages for ≥20 and ≥10 μg/dL than for the Lanphear and Jordan studies, which didn’t even calculate levels ≥5 μg/dL. With increasing concerns about adverse effects at BLLs <10 μg/dL there may be more focus on interventions at lower BLLs. Paradoxically, with lower levels it becomes harder to demonstrate differences between populations and to document change in BLL as a health outcome of preventive interventions. Future studies may need to utilize other health outcomes or non-health outcomes such as changes in environment, knowledge or behavior and should collect information on non-housing-based lead exposure sources. 

We did examine other sources of lead exposure, and except for a relatively high percentage of parents (39.8%) with work experience in industries with potential lead exposures, our cohort did not have non-housing sources of exposure. Our study did document positive changes, such as increased parental knowledge and cleaning activity (representing a behavioral change) and decreased home lead dust levels, which seemed to result from our interventions. 

Study limitations include lack of detailed knowledge about the comparison group of children, the low number of families following through with the process for study-provided lead remediation work, and the lower number of families completing all three home visits through study attrition. 

A higher participation rate for remediation work and/or study visits might have resulted in lower BLLs for the LSH Study cohort. A recent study looking at aggregate remediation efforts in Chicago attempted to derive estimates of the effect of remediation, working with census tract level data [17]. Since percentage of houses remediated is an indicator of old, poorly-maintained housing, this kind of research has to deal with numerous confounders, which can only be partially controlled even with individual-level data, let alone census tract-level data. After adjustment for confounding, Jones confirmed that remediation appears to lower the prevalence of lead poisoning (defined as an elevated BLL of ≥10 μg/dL). He also found that, citywide, 2.5 cases of lead poisoning were adverted for every housing unit remediated. Lastly, he found that benefits from decreased prevalence, such as increased expected lifetime earnings and decreased medical expenditures for poisoned children, were valued at 2–20 times the estimated costs of the home remediation [17]. 

Despite the recognized benefits of lead remediation observed in the Jones study and by others, the practical experience of our LSH study team was that it was apparently difficult for study families to commit to receiving such remediation work. The families that remained in the study may represent those who were more likely to maintain a cleaner home and take actions to prevent lead exposure. Those children that received the blood lead testing around two years of age might have had more proactive parents or health care providers (although a blood lead screen is recommended for all two-year-old children in Philadelphia), or higher levels around one year prompting parents to request repeat testing. 

Conducting studies in poor urban communities is challenging as many families have competing interests, many of them related to obtaining basic life services such as housing, food, employment, child care, *etc*. Therefore, imposing another commitment (such as a study protocol) can be too much for some families to absorb into their lives and adhere to. We did not randomize a group to receive no intervention, but to two different intervention levels with use of a similar group of children for comparison, due to concerns that IRB approval might not be forthcoming for a protocol utilizing a group not receiving interventions. Strengths of the study include obtaining a large N for both the cohort and comparison groups and a relatively long study intervention period. A relatively large number from both groups had blood lead levels available for both the one and two year analysis, although these numbers did drop for the two year analysis.

## 5. Conclusions

A comparison of geometric mean blood lead levels and percentages above several BLL cut points drawn around age two years in a group of urban newborns benefitting from educational and environmental interventions *versus* a group of similar urban children did not yield statistically significant differences. Both groups had relatively lower lead levels when compared to historical cohort groups and this may reflect a continuing downward trend in BLLs in U.S. children. Documenting significant changes in blood lead levels as a result of interventions may be increasingly difficult due to this trend and other outcomes of primary prevention may need to be studied. The study interventions did result in benefits to the families such as an increase in parental knowledge about lead exposure/poisoning prevention and in-home wet cleaning activity, and a decrease in lead dust levels in study homes. 

## Appendix

**Table:** Regression 1: Controlled for age and insurance type

Coefficients ^a^						
**Model**		**Unstandardized Coefficients**		**Standardized Coefficients**	**t**	***p* value**
		B	Std. Error	Beta		
1	(Constant)	0.825	0.154		5.356	0.000
	Group	−0.032	0.040	−0.042	−0.806	0.421
	Age at 2 year BLL	−0.056	0.055	−0.053	−1.018	0.309
	Insurance	−0.086	0.032	−0.137	−2.732	0.007

^a^ Dependent Variable: Logarithm (base 10) of BLL at year 2; Key: Group is coded: 1 = LSH, 2 = control; Age is in years, with 2 decimals places; Insurance is 1 = Medicaid insurance 2 = private health insurance; Regression equation: Log (Y-2 BLL) = −0.042*group -0.053*age −0.137*insurance. All standardized weights.

**Table:** Regression 2: Controlled for age, insurance type, and year 1 BLL

Coefficients ^a^						
**Model**		**Unstandardized Coefficients**		**Standardized Coefficients**	**t**	***p* value**
		B	Std. Error	Beta		
1	(Constant)	0.553	0.148		3.730	0.000
	Group	−0.009	0.037	−0.012	−0.239	0.812
	Age at 2 year BLL	−0.057	0.054	−0.053	−1.062	0.289
	Year 1 BLL (log base 10)	0.420	0.044	0.456	9.459	0.000
	Insurance	−0.051	0.030	−0.081	−1.678	0.094

^a^ Dependent Variable: Logarithm (Base 10) of year 2 BLL; Key: Group is coded: 1 = LSH, 2 = control; Age is in years, with 2 decimals places; Insurance is 1 = Medicaid insurance 2 = private health insurance; Regression equation: Log (Y-2 BLL) = −0.012*group −0.053*age + 0.456*Y-1 BLL − 0.081*insurance. All standardized weights.
